# Characterization and antibiogram of bacterial isolates from diseased farmed Nile tilapia in Beheira governorate, Egypt

**DOI:** 10.1186/s12917-025-05227-4

**Published:** 2026-01-10

**Authors:** Merna M. A. Hassan, Riad H. Khalil, Mahmoud M. Abotaleb, Mahmoud T. Amer, Hany M. R. Abdel-Latif

**Affiliations:** 1 Department of Poultry and Fish Diseases, Faculty of Veterinary Medicine, Alexandria University, Alexandria, 22758 Egypt; 2 Central Laboratory for Evaluation of Veterinary Biologics, Agriculture Research Center (ARC), Cairo, Egypt

**Keywords:** Distribution, Antimicrobial resistance, Nile tilapia, Mortalities, Bacterial pathogens

## Abstract

This study aimed to isolate, identify, and describe the bacteria isolated from mortality events occurred among pond-farmed Nile tilapia in different farms at Edku, Beheira province, Egypt, with special emphasis on their antimicrobial resistance profile. Specimens were collected from six private farms that experienced mortality outbreaks that occurred throughout the year between August 2023 and 2024. Clinical and postmortem examinations uncovered characteristic signs of bacterial septicemia. Forty-three bacterial isolates were recovered from the examined moribund and recently dead specimens. Based on the genetic data and evolutionary relationships derived from the 16S ribosomal RNA gene, six different isolates were resolved as *Streptococcus agalactiae* (46.5%), *Vibrio alginolyticus* (11.6%), *Vibrio campbellii* (14%), *Vibrio owensii* (9.3%), *Aeromonas veronii* (11.6%), and *Enterococcus faecalis* (7%). The antibiogram results revealed varying susceptibility patterns among the isolates. *V. alginolyticus* showed intermediate susceptibility to ciprofloxacin, while *V. campbellii* was only moderately sensitive to oxytetracycline. Of particular concern, *A. veronii* was found to be resistant to all tested antimicrobials. However, *E. faecalis* displayed sensitivity to ciprofloxacin, making it a potential drug of choice for treating diseased tilapia; this isolate was also moderately susceptible to oxytetracycline, erythromycin, and kanamycin. Finally, *S. agalactiae* exhibited intermediate susceptibility only to amoxycillin/clavulanic acid, oxytetracycline, and ciprofloxacin. Our findings clearly demonstrate that careless antibiotic administration in fish farming drives the emergence of antimicrobial resistance. Therefore, proactive planning is necessary, encompassing comprehensive surveillance, and the establishment of strict control and prevention measures to curb bacterial spread and safeguard productivity in Tilapia aquaculture.

**Supplementary Information:**

The online version contains supplementary material available at 10.1186/s12917-025-05227-4.

## Introduction

Egypt is the largest aquaculture producer in Africa with a promise for expansion through transitioning from traditional to intensive farming systems. In Egypt, Nile tilapia represents the key farmed species that places it as one of the top countries in tilapia aquaculture production [1, 2]. Infectious diseases have been a long-standing challenge for the growth and sustainability of fish farming, especially of the bacterial origin resulting in huge economic losses [3–5]. Egyptian tilapia aquaculture has recently been hampered by a series of unusual mass mortalities, with a significant increase during the summer season [6]. Those authors surveyed 113 tilapia farms in several localities in Kafr El Sheikh, Beheira, Sharqia, and Faiyum governorates and declared that source and quality of water were the most significant contributing factors to both emergence and intensity of summer tilapia mortalities.

According to a recent review, it was found that a variety of bacterial infections have been identified as the cause of such frequent mortality events, which were linked to water temperature, high nutrient concentrations, and fish densities [7]. Zaheen et al. [8] also confirmed that the bacterial disease has been resulted from multi-factorial condition influenced by interaction between the bacteria, its host, and aquatic environment. Several other studies revealed that the prevalence of bacterial fish diseases is triggered by numerous stressors such as the water temperature over 28 °C, unionized ammonia exceed 0.2 mg/L, pH > 8.5, high stocking density and bad managemental practices [9]. Similarly, El-Son et al. [10] elucidated that poor water quality as decreased DO levels, elevated levels of unionized ammonia and nitrite as well as increased heavy metal contents were identified as stress triggers influencing fish health and increased their vulnerability to bacterial infections.

Bacterial fish pathogens have multiple virulence mechanisms that play a critical role in their pathogenesis [11]. Abdallah et al. [12] reported a mass mortality case of *Streptococcus agalactiae* infecting cultured *O. niloticus* in Assiut, Egypt. Huicab-Pech et al. [13] clarified that tilapia is susceptible to multiple pathogenic bacteria under stress conditions. Motile Aeromonas septicemia (MAS), Streptococcosis, and Vibriosis which are among bacterial diseases causing summer mortalities in tilapia farms in Egypt. A disease outbreak has also been reported by *Aeromonas* spp., *Vibrio alginolyticus*, and *Enterococcus faecalis* infection in Nile tilapia and African catfish in Manzala, Egypt [14, 15]. Those authors declared that inferior water quality such as high levels of ammonia, nitrite, and nitrate are the prominent cause of high mortalities. Abu-Elala et al. [16] identified three other bacterial pathogens including *E. faecalis*, *S. agalactiae*, and *Lactococcus garvieae* as the most significant pathogens that severely impact tilapia aquaculture. A study conducted by Elgohary et al. [17], found that *E. faecalis* and *Aerococcus viridans* have emerged as important fish diseases affecting Tilapia fish farms in El Sharkia and El Fayoum provinces. Likewise, Osman et al. [18] illustrated that *Streptococcus* sp., *E. faecalis* and *L. garvieae* were considered as new candidate bacterial pathogens in tilapia aquaculture.

Antibiotics are commonly used to manage and cure bacterial infections in fish [19]. Nevertheless, misusing these medications significantly drives the evolution of antibiotic-resistant bacteria, which can then pass their resistance genes to other bacterial types [20]. This ultimately reduces the effectiveness of these drugs for both aquatic life and human health [21, 22]. Therefore, the purpose of study to describe the clinical picture, and antimicrobial profile of the most common bacterial pathogens isolated from pond-reared Nile tilapia with health disorders in some Egyptian farms within Beheira governorate, Egypt.

## Materials and methods

## Sampling

Sixty adult diseased *O. niloticus* of different body weights (80 ± 20 g) and lengths were collected from six private farms located in different localities at Edku, Beheira province, Egypt throughout a year between August 2023 and 2024. Fish were stocked at a density of 20,000 to 25.000 fish per hectare. The main water supply of these farms is agricultural drainage from Edku. We observed poor biosecurity and managemental practices within the surveyed tilapia farms throughout the production cycle. Freshly dead samples only were taken and then were packed in an icebox while the moribund and alive fish were kept in well-aerated plastic bags. Clinical, necropsy, postmortem (PM) and bacteriological examinations were conducted using the methods outlined by Austin and Austin [23]. Fish samples showing septicemic signs were examined. The necropsy of the naturally infected tilapia fish was performed under completely sterile conditions.

## Bacterial isolation and identification

### Bacterial isolation

Before fish opening, the skin of each fish sample was wiped with 70% ethyl alcohol for surface disinfection. The liver, kidney, and spleen of the diseased tilapia were sampled for microbiological isolation by direct culturing onto Tryptic Soy Broth (TSB; Difco™) for pre-enrichment, incubated overnight at 30 °C. Sterile loopfuls were inoculated onto Tryptic Soya Agar (TSA; Difco™), Brain Heart Infusion Agar (BHIA; Difco™), and Thiosulphate Citrate Bile Salts Sucrose agar (TCBS; HiMedia™) followed by incubation at 30 °C for 24–48 h. The dominant bacterial colonies were selected and sub-cultured. After incubation, bacterial colonies were picked and streaked onto TSA supplemented with 5% sheep blood to examine the hemolytic activity. Consequently, the pure stock isolates were preserved at − 80 °C with 20% (v/v) glycerol for further biochemical and molecular examinations.

### Morphological and biochemical characterizations

Gram staining, culture characteristics, hemolytic activity, motility, oxidase test, catalase test, and salt-tolerant test (NaCl 2%, 4%, 6%, 8%, and 10%) were performed to characterize the isolated bacteria. The results were recorded and interpreted based on the identification manual of bacteria from fish [24]. Successively, the VITEK examination was performed to evaluate the phenotypic characteristics of recovered isolates [25]. Pure bacterial colonies were streaked over TSA and incubated at 30 °C for 24 h to be identified by VITEK 2 compact analyzer (BioMérieux, France) following the manufacturer’s instructions.

### Molecular identification and characterization

Molecular examination was carried out on six different randomly selected bacterial isolates. In brief, the bacterial DNA was extracted from 24 h growing bacterial isolates in TSB using QIAamp DNA mini kit, Catalogue No. 51,304, USA following the manufacturer’s protocol. Partial fragments of 16S rRNA gene were amplified using *Aeromonas* 16S rRNA (953 bp), *Vibrio* 16S rRNA (663 bp), *S. agalactiae* 16S rRNA (405 bp), and universal 16S bacterial primers (1485 bp) for *S. agalactiae* and other bacterial species (Table 1).

**Table 1:** Oligonucleotide primers that were used for bacterial identification in this study

Bacterial species	Target genes	Product size (bp)	Primer sequences	References
*V. alginolyticus*	*Vibrio* 16S rRNA	663	CGGTGAAATGCGTAGAGAT	[26]
*V. campbellii*			TTACTAGCGATTCCGAGTTC	
*V. owensii*				
*A. veronii*	*Aeromonas* 16S rRNA	953	CTACTTTTGCCGGCGAGCGG	[27]
			TGATTCCCGAAGGCACTCCC	
*E. faecalis*	Bacterial 16S rRNA	1485	AGAGTTTGATCMTGGCTCAG	[28]
			TACGGYTACCTTGTTACGACTT	
*S. agalactiae*	Bacterial 16S rRNA			
	*S. agalactiae* 16S rRNA	405	CGCTGAGGTTTGGTGTTTACA	[29]
			CACTCCTACCAACGTTCTTC	

Preparation of PCR reaction mixture was performed using 2X Dream Taq Green master mix kit and the reactions were executed in a final volume of 25 µL in a DNA thermal cycler (T3 Thermal cycler, Biometra). The amplification conditions were detailed in our recently published research [1]. The PCR products were analyzed by electrophoresis in 1.5% agarose gel, stained and photographed by gel documentation system (Alpha Innotech). The PCR products were purified using QIA quick PCR Purification kit (QIAGEN, USA) according to the manufacturer’s protocol.

### Virulence genes detection

The presence of key virulence genes in the genome of the six identified bacterial isolates was detected by PCR. *V. alginolyticus* was analyzed for thermostable direct hemolysin (*tdh*), and thermostable direct hemolysin-related hemolysin (*trh*). *V. campbellii* and *V. owensii* were examined for thermolabile hemolysin (*tlh*). *A. veronii* was tested for the presence of the genes encoding cytotoxic enterotoxins (*act*) and aerolysin (*aerA*). *E. faecalis* was tested for gelatinase (*gelE*), and cytolysin (*cylA*). *S. agalactiae* was screened for β-hemolysincytolysin (*cylE*), and hyaluronidase (*hyl*) to determine the relationship between the existence of these genes and the virulence of retrieved bacteria in cultured *O. niloticus*. Amplicon genomic DNA was extracted using a method analogous to the protocol established for bacterial identification. Primers used and PCR conditions are summarized in Table S2 (*Supplementary Material*).

## Sequencing and phylogenetic analysis

The resulting purified DNA was subjected to sequencing using an Applied Biosystems 3130 automated DNA Sequencer (ABI 3130, USA). This was accomplished via a cycle sequencing reaction using the BigDye Terminator v3.1 ready reaction kit (Perkin-Elmer/Applied Biosystems, Cat. No. 4336817). The obtained 16S rRNA gene sequences from the bacterial isolates were then cross-referenced and confirmed. This identification process involved comparing the sequences’ homology against existing entries in the GenBank database utilizing the Basic Local Alignment Search Tool (BLAST analysis). The obtained sequential data was aligned by CLUSTAL W in MEGA 12.0 software. The aligned sequences were analyzed to suggest possible evolutionary relationships and to construct the phylogenetic tree via the maximum likelihood methods with 1000 bootstrapping test [30].

## Antimicrobial susceptibility testing (AST)

Before testing, the Quality Control (QC) strains were used for ensuring the accuracy, reliability, and reproducibility of AST as suggested by CLSI (Clinical and Laboratory Standards Institute) [31]. The antibiogram was executed for the identified bacterial isolates to nine types of commercial antimicrobial discs, namely ampicillin (AMP) 10 µg, penicillin (P) 10 µg, amoxycillin/clavulanic acid (AMC) 20/10 µg, oxytetracycline (O) 30 µg, erythromycin (E) 15 µg, kanamycin (K) 30 µg, novobiocin (NV) 30 µg, ciprofloxacin (CIP) 5 µg, and trimethoprim/sulfamethoxazole (COT) 25 µg placed on Mueller-Hinton agar (Oxoid™) using the disc diffusion method. The findings were measured as inhibition zone (clear zone around the antibiotic disc) and interpreted based on susceptible (S), intermediate (I), and resistance (R) as previously described by CLSI [31].

## Results

### Clinical signs and necropsy findings

**Fig. 1:**
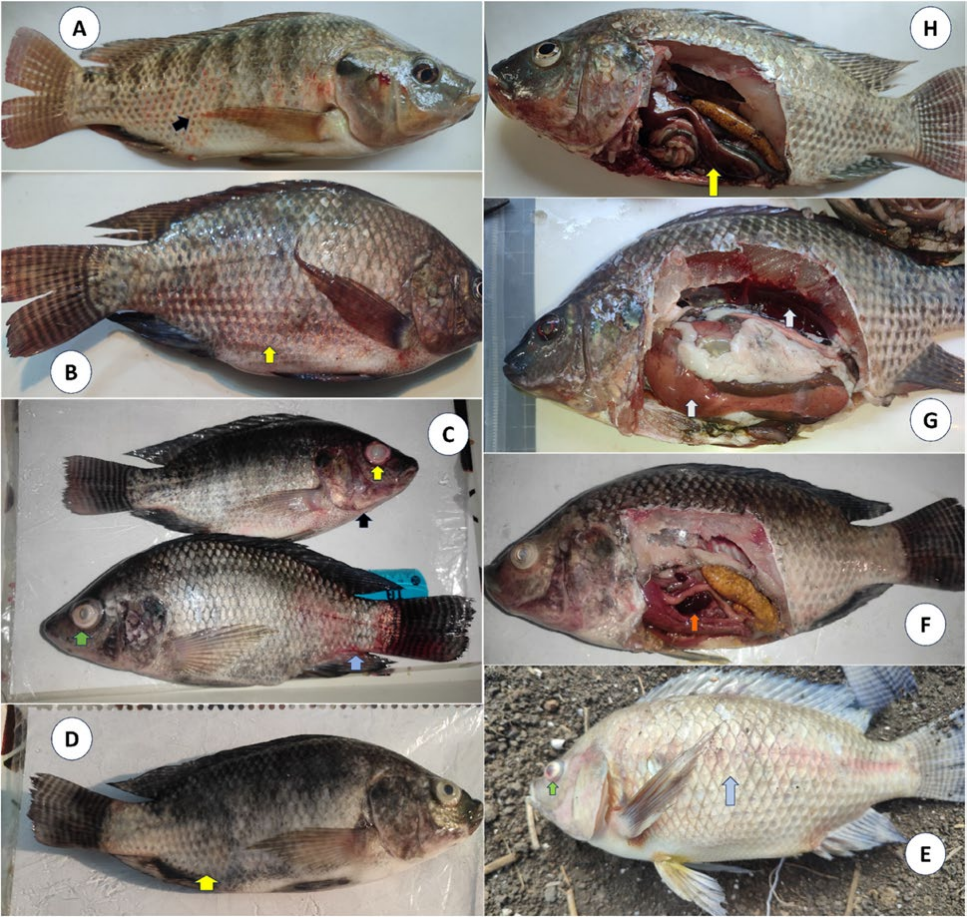
Clinical signs and gross lesions of diseased Nile tilapia. Diseased fish showed several external signs including erythema (**A**; black arrow) and hemorrhagic patches over the abdomen (**B**; yellow arrow), corneal opacity (green arrow) associated with hemorrhage at the caudal peduncle (blue arrow), unilateral exophthalmia and opaqueness on the eye (yellow arrow) associated with severe opercular hemorrhages (black arrow) (**C**), detached scales (**D**; yellow arrow), abdominal dropsy, exophthalmia (green arrow), dermal hemorrhages (blue arrow) (**E**). The gross lesions appear as congested spleen (**F**; orange arrow), mottled liver and congested kidneys (**G**; white arrows), and congested liver (**H**; yellow arrow)

In this study, the diseased Nile tilapia generally exhibited lethargy, sluggish movement, anorexia, and certain cases showed erratic swimming. Most infected tilapia fish showed septicemic clinical signs. As shown in Fig. 1, the diseased fish showed several external signs including erythema and hemorrhagic patches over the abdomen, corneal opacity associated with hemorrhage at the caudal peduncle, unilateral exophthalmia and opaqueness on the eye associated with severe opercular hemorrhages, detached scales, abdominal dropsy with protruded vent, exophthalmia, dermal hemorrhages. The gross lesions appear as congested spleen, mottled liver and congested kidneys, and liver with accumulation of serous ascitic blood-tinged fluid in the abdominal cavity.

### Phenotypic identification and biochemicals characterization

A total of 43 (n) bacterial isolates were retrieved and identified using conventional and biochemical tests, as shown in Table 2. The laboratory bacteriological examination detected three genera: *Streptococcus* spp., *Vibrio* spp., *Aeromonas* spp., and *Enterococcus* spp. Initially, *Vibrio* isolates produced characteristic yellow, green, or yellow-greenish colonies on their selective culture media (TCBS). On sheep blood agar, *V. alginolyticus* produced β-hemolytic colonies, while *V. campbellii*, *V. owensii*, and *A. veronii* showed no hemolysis. Moreover, *E. faecalis* showed α-hemolytic activity whereas *S. agalactiae* typically formed gray-white colonies with narrow zone of beta hemolysis.

**Table 2:** Microbiological and biochemical characteristics of the retrieved bacterial isolates

Bacterial isolates	*V. alginolyticus* (*n* = 5)	V. campbellii (*n* = 6)	*V. owensii* (*n* = 4)	*A. veronii* (*n* = 5)	*E. faecalis* (*n* = 3)	*Str. agalactiae* (*n* = 20)
Gram stain	-ve	-ve	-ve	-ve	+ve	+ve
Shape	curved rods	curved rods	curved rods	short rods	cocci	cocci
Motility	Motile	Motile	Motile	Motile	Non-motile	Non-motile
Swarming activity	+	+	+	+	-	-
Oxidase	+	+	+	+	-	-
Catalase	+	+	+	+	-	-
Hemolysis on blood agar	β	γ	γ	γ	α	β
TCBS medium	large convex, yellow-colored colonies	Round green colonies	circular yellowish-green colonies	ND	ND	ND
Growth at % NaCl						
0%	+	+	+	+	+	+
2%	+	+	+	+	+	+
4%	+	+	+	+	+	+
6%	+	+	+	+	+	+
8%	+	+	+	+	+	+
10%	-	-	-	-	-	-

*+ ve* Positive, *-ve* Negative, *ND* Not Done, *α* Alpha hemolysis, *β* Beta hemolysis, γ Gamma hemolysis

The Gram stain grouped the isolates into (23) Gram-positive and (20) Gram-negative collected from moribund tilapia. The *Vibrio* spp. was Gram-negative, curved or comma-shaped rods, while *A. veronii* were Gram-negative, short, rod-shaped bacteria. On the other hand, *E. faecalis* were defined as Gram-positive cocci arranged in clusters, whereas *S. agalactiae* were Gram-positive cocci that organize into marked chains. All retrieved bacteria could survive in varying salt concentrations, up to 8% NaCl. For oxidase and catalase tests, *Vibrio* and *Aeromonas* spp. demonstrated positive results, while *Enterococcus* and *Streptococcus* spp. showed negative reactions.

Based on the results of VITEK examination, purified isolates were tested for different test parameters as detailed in *Supplementary Material* (*Tables S2-S4*). It was reported that some isolates belonged to either *Vibrio* and Aeromonads or *Enterococcus* spp. The tested *V. alginolyticus* were identified based upon positive reaction to glucose, maltose, and mannitol utilization tests. The isolates were typically negative for O/129 resistance, H_2_S production, citrate utilization, and urea hydrolysis. These results confirm the identity of *V. alginolyticus*, which is consistent with the expected phenotypic characteristics. VITEK identification only validated *Aeromonas* isolates at the genus level with 50% probability. Retrieved *Aeromonas* spp. produced positive reactions to glucose, maltose, and mannose utilizations, O/129 resistance, and β-galactosidase production. However, it tested negative for citrate utilization, urease, and H_2_S production. In addition, *E. faecalis* could utilize various sugars including galactose, mannose, maltose, ribose, and N-acetylglucosamine, while also detecting negative for raffinose and urease tests. The significant findings facilitate the differentiation of these isolates from other bacterial species.

### Molecular characterization of isolated bacteria based on 16S rRNA sequence

Six isolates of three different *Vibrio* spp., one isolate of *A. veronii*, one isolate of *S. agalactiae*, and one isolate of *E. faecalis* were positive for 16S rRNA gene primers amplified the expected conserved region at 663, 953, 405, and 1485 bp, respectively.

**Fig. 2:**
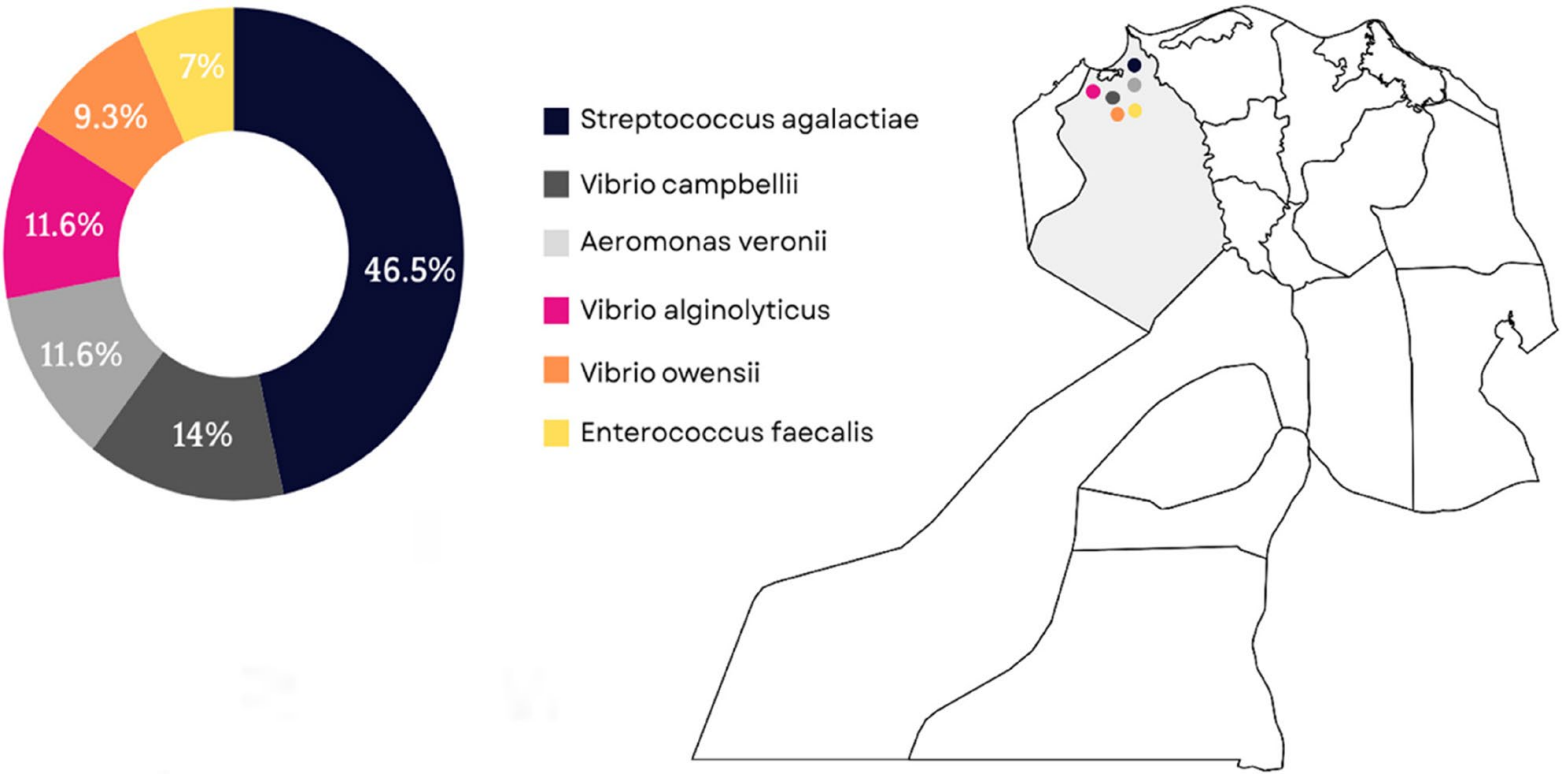
Egypt map, grey zone is Edku, Beheira province from which diseased Nile tilapia were sampled with the prevalence percentage of isolated bacteria

According to the culture characters, biochemical, and molecular profile, bacterial isolates were identified as *S. agalactiae* (46.5%), *V. alginolyticus* (11.6%), *V. campbellii* (14%), *V. owensii* (9.3%), *A. veronii* (11.6%), and *E. faecalis* (7%) *(*Fig. 2*).* The presence of virulence-associated genes in the identified bacteria isolated from diseased tilapia samples is shown in Figures S1-S3 (*Supplementary Material*). *V. alginolyticus* strain harbored *tdh* and *trh* genes whereas, *tlh* gene was detected in *V. campbellii* and *V. owensii* isolates. *A. veronii* strain was positive for presence of *act* and *aerA* genes. *E. faecalis* strain was negative for *cylA* gene, while it possessed the *gelE* gene. In addition, *cylE* gene was identified in *S. agalactiae* strain, while it tested negative for *hyl* gene.

### Results of sequencing and phylogenetic analysis

The obtained nucleotide sequences showed identity with the following strains: *V. alginolyticus* (strain VA1), *V. campbellii* (strain VC2), *V. owensii* (strain VO3), *A. veronii* (strain AV2), *E. faecalis* (strain EF2), and *S. agalactiae* (strain SA1) deposited in GenBank under accession numbers; *PV016851*, *PV016852*, *PV016853*, *PV018986*, *PV013414*, and *PV010354*, respectively. The BLAST result of *Vibrios*, the 16S rRNA sequence of *V. alginolyticus* shared 100% identity with *V. alginolyticus* PQ821160 from India, PV833938 from India, PV639166 from Italy, and PV639167 from Italy. The retrieved sequence of *V. campbellii* showed 100% similarity with *V. campbellii* OM891761 from Nigeria and ON437449 from China. The similarity was 99.55% with *V. campbellii* OP343775 from Spain and 99.85% with *V. campbellii* ON405294 from China. Furthermore, *V. owensii* sequence showed 100% identity with *V. owensii* PQ669449 from India, OR262801 from China, PP515033 from China, and PV596532 from Philippines.

**Fig. 3:**
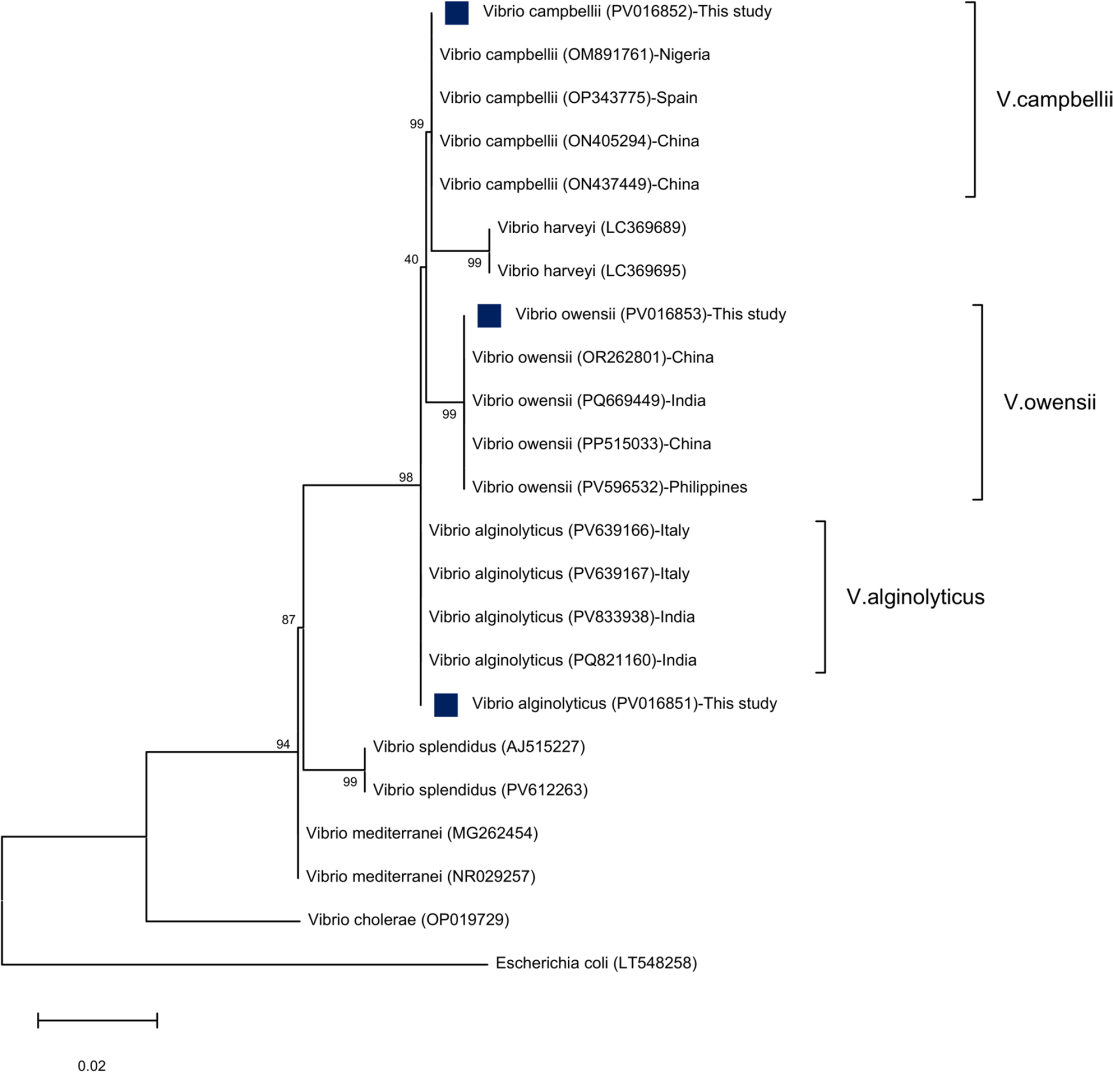
Phylogenetic tree based on 16S rRNA partial sequences showing relationships of three isolated *Vibrio* strains; *V. alginolyticus*, *V. campbellii*, and *V. owensii* with other described closely related *Vibrio* species and out-group *E. coli* retrieved from GenBank database. Phylogenetic analyses were conducted with MEGA12.0, using the maximum likelihood method with the Jukes-Cantor model allowing sites to be evolutionary invariable (I). Percentage bootstrap values (1000 replicates) are shown at each branch point. The scale bar 0.02 represents substitutions per nucleotide position. The blue squared dots represent the studied isolates

A maximum-likelihood based phylogenetic tree revealed that three representative *Vibrio* spp. combined with *V. harveyi*, *V. splendidus*, *V. mediterranei*, and *V. cholera* showing evolutionary relatedness with 99% bootstrapping values of *V. campbellii* and *V. owensii* in addition to 98% bootstrap value of *V. alginolyticus* (Fig. 3).

**Fig. 4:**
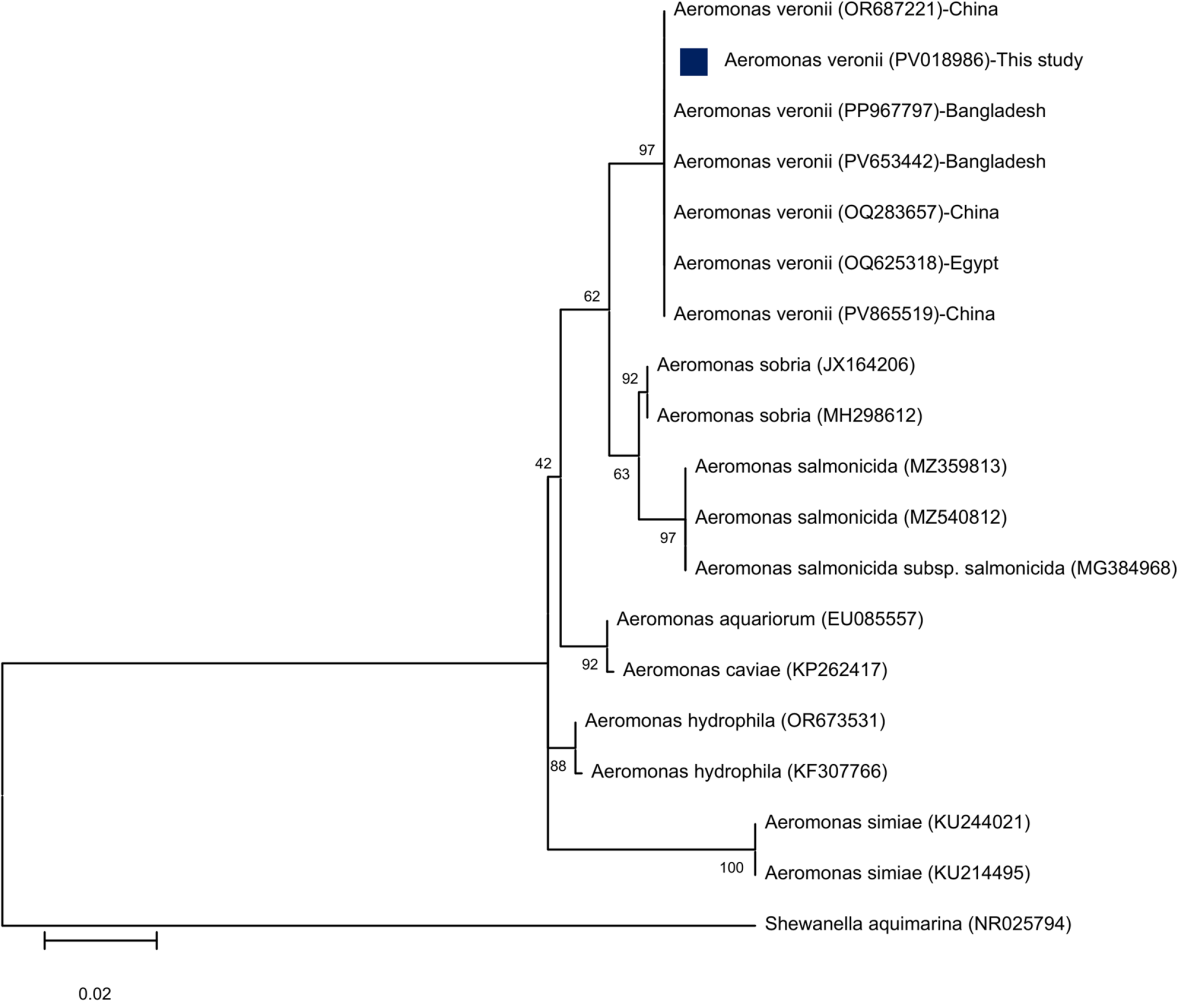
The Phylogenetic tree showing relationship of 16S rRNA partial sequence of *A. veronii* and the 16S rRNA gene of other reported closely related Aeromonad species and with *Shewanella aquimarina* was selected as out-group from GenBank. Phylogenetic analyses were conducted with MEGA12.0, using the maximum likelihood method with the Hasegawa-Kishino-Yano model with Gamma distribution (G). Percentage bootstrap values (1000 replicates) are shown at each branch point. The scale bar 0.02 represents substitutions per nucleotide position. The blue squared dot represents the studied isolate

Blast analysis of *A. veronii* sequence shared 100% identity with *A. veronii* OR687221 from China, PP967797 from Bangladesh, PV653442 from Bangladesh, OQ283657 from China, OQ625318 from Egypt, and PV865519 from China. Another phylogenetic tree was drawn using maximum-likelihood method showing that the *A. veronii* isolates were separated from other *Aeromonas* spp. including *A. sobria*, *A. caviae*, *A. hydrophila*, *A. aquariorum*, *A. simiae*, and *A. salmonicida* with a bootstrap value of 97% as described in (Fig. 4*)*.

**Fig. 5:**
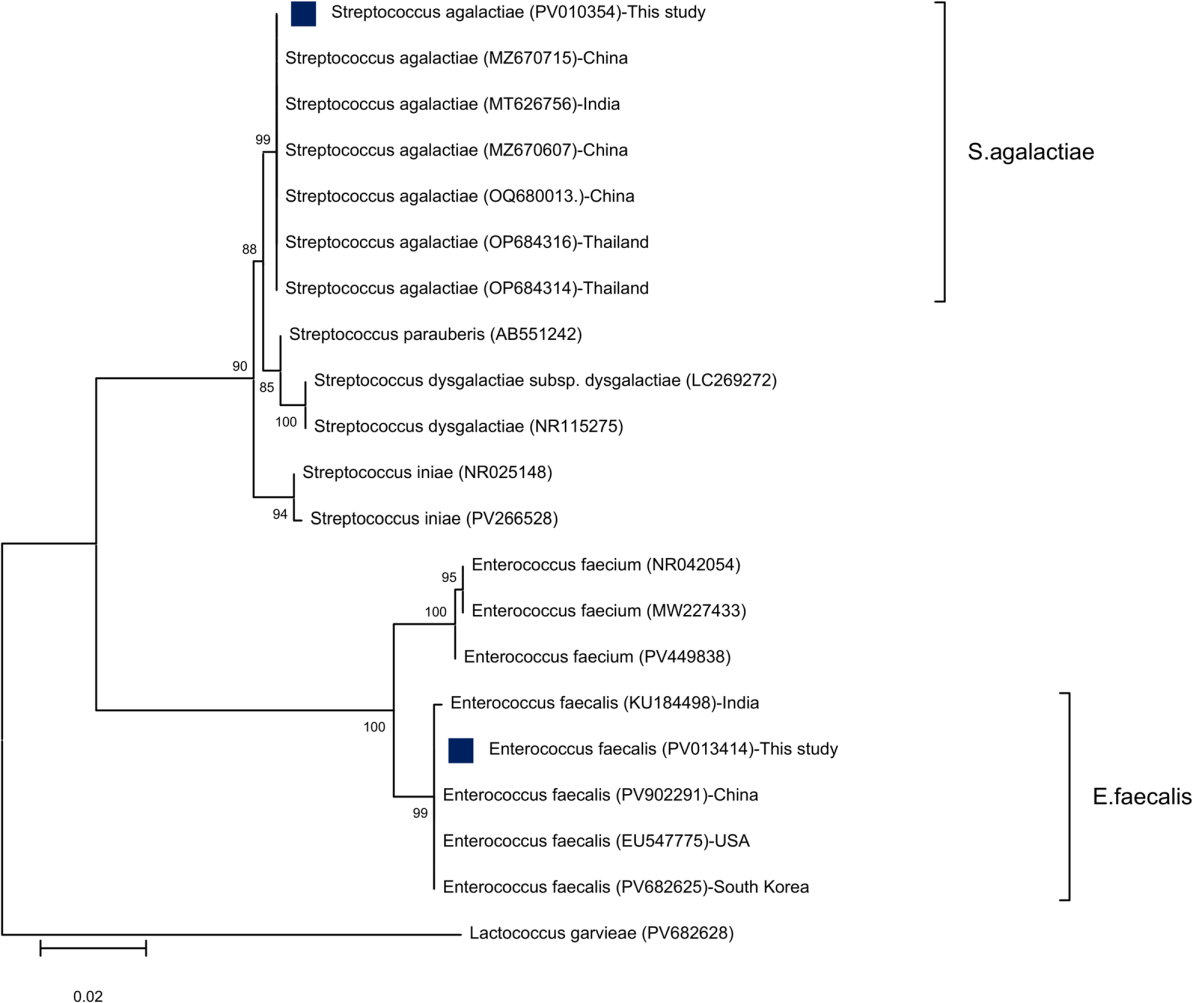
Phylogenetic tree based on 16S rRNA partial sequences showing relationships of *S. agalactaie* and *E. faecalis* with other described closely related species and out-group *Lactococcus garvieae* retrieved from GenBank database. The Maximum likelihood method was used with the Kimura 2-parameter model with Invariant sites (I). The support level in percentage, after 1000 repetitions, is indicated next to each branch. The scale bar 0.02 represents substitutions per nucleotide position. The blue squared dots represent the studied isolates

Similarly, the blast result of sequenced *E. faecalis* shared 100% identity with *E. faecalis* KU184498 from India. The similarity was 99.86% with *E. faecalis* PV902291 from China, EU547775 from USA, and PV682625 from South Korea. In addition, sequenced *S. agalactiae* revealed 100% identity with *S. agalactiae* MZ670715 from China, MT626756 from India, MZ670607 from China, OQ680013 from China, OP684316 from Thailand, and OP684314 from Thailand. A phylogenetic tree based on maximum likelihood showed two main lineages. The first clade included *S. agalactiae* isolate of this study accompanied by *S. parauberis*, *S. dysgalactiae*, and *S. iniae* with a 99% bootstrap value. The second subclade contained the isolated *E. faecalis* strain with other similar species, exhibiting a 99% bootstrap value (Fig. 5).

### Distribution and diversity of the bacterial isolates

According to Table 3, forty-three (43) bacterial isolates were retrieved from the liver, kidney, and spleen of naturally infected fish. From which, *S. agalactiae* (20) was the most prevalent bacterial species followed by *V. campbellii* (6), *A. veronii* (5), *V. alginolyticus* (5), *V. owensii* (4), and *E. faecalis* (3). The distribution of these bacteria in different tissues of infected fish was equally in liver and kidney (15) followed by spleen (13).

**Table 3:** Details of fish sampling from beheira province, Egypt

Date of collection	Culture system	No. of isolates	Tissue samples		
			Liver	Kidney	Spleen
August 2023	F1-CP	9	1	5	3
October 2023	F2-EP	5	3	1	1
October 2023	F3-EP	6	3	1	2
October 2023	F3-EP	4	2	1	1
October 2023	F4-CP	5	2	1	2
November 2023	F5-EP	3	2	1	0
August 2024	F6-EP	11	2	5	4
Total	6	43	15	15	13

*F* Farm, *EP* Earthen Pond, *CP* Cement Pond

### AST results

Most bacterial isolates showed resistance to most tested antibiotics as summarized in Table 4. Three *Vibrionaceae* isolates revealed complete resistance against ampicillin, penicillin, amoxycillin/clavulanic acid, erythromycin, kanamycin, novobiocin, and trimethoprim/sulfamethoxazole. Conversely, *V. alginolyticus* exhibited intermediate sensitivity to ciprofloxacin, whereas *V. campbellii* was moderately susceptible to oxytetracycline. The unexpected finding revealed that *A. veronii* was completely resistant to all antimicrobial agents. In contrast, *E. faecalis* was susceptible to ciprofloxacin, while this isolate was intermediate-sensitive to oxytetracycline, erythromycin, and kanamycin. Intermediate antibiotic susceptibility of *S. agalactiae* was only shown to amoxycillin/clavulanic acid, oxytetracycline, and ciprofloxacin.

**Table 4:** The AST of the retrieved bacterial isolates by disc diffusion assay

The tested antimicrobials	Class	Conc. (µg)	Bacterial isolates					
			VA1	VC2	VO3	AV2	EF2	SA1
Ampicillin (AMP)	Beta lactam	10	0(R)	0(R)	0(R)	0(R)	10(R)	0(R)
Penicillin (P)	Beta lactam	10	0(R)	0(R)	0(R)	0(R)	8(R)	0(R)
Amoxycillin/Clavulanic acid (AMC)	Beta lactam	20/10	9(R)	10(R)	0(R)	0(R)	13(R)	15(I)
Oxytetracycline (O)	Tetracycline	30	8(R)	21(I)	0(R)	0(R)	20(I)	25(I)
Erythromycin (E)	Macrolides	15	0(R)	0(R)	0(R)	0(R)	14(I)	0(R)
Kanamycin (K)	Aminoglycosides	30	0(R)	0(R)	0(R)	0(R)	16(I)	0(R)
Novobiocin (NV)	Aminocoumarin	30	9(R)	0(R)	0(R)	0(R)	15(R)	0(R)
Ciprofloxacin (CIP)	Fluoroquinolones	5	18(I)	0(R)	0(R)	0(R)	23(S)	20(I)
Trimethoprim/Sulfamethoxazole (COT)	Diaminopyrimidine + Sulfonamides	25	0(R)	0(R)	0(R)	0(R)	0(R)	0(R)

Zone of inhibition measured = mm

*R* Resistant, *I* Intermediate, *S* Sensitive

(VA1): *V. alginolyticus*; (VC2): *V. campbellii*; (VO3): *V. owensii*; (AV2): *A. veronii*; (EF2): *E. faecalis*; (SA1): *Str. agalactiae*

## Discussion

The present study depicted the bacterial pathogens encountered in heavy mortality events among pond-farmed Nile tilapia at Beheira Province, Egypt. The presumptive identification based on the clinical examination of the diseased fish revealed clinical signs like those reported in septicemic bacterial infections. These findings were like those found in previous reports in Nile tilapia [32, 33].

The PCR-based molecular analysis plays an important part in identifying new organisms or confirming morphological and biochemical features of fish-associated pathogens [34]. The 16S rRNA sequence testing confirmed the suspected bacterial isolates collected from moribund tilapia. Blast results and phylogenetic analysis based mainly on the 16S rRNA gene sequences of is important for diagnosis of the retrieved bacterial pathogens [10]. The 16S rRNA gene sequences of recovered isolates were almost identical to those of their related species on GenBank database, with a similarity greater than 99%. This gene facilitates classifying those bacteria into different genera, including *Vibrionaceae*, aeromonads, enterococci, and *Streptococcus* sp [35–38]. In our study, *V. alginolyticus* (PV016851), *V. campbellii* (PV016852), *V. owensii* (PV016853), *A. veronii* (PV018986), *E. faecalis* (PV013414), and *S. agalactiae* (PV010354) were identified and isolated from diseased tilapia.

*A. veronii* was primarily misidentified as *A. sobria* using VITEK2 with 50% probability, while the sequencing confirmed its identity. In this essence, Abdelsalam et al. [39] confirmed that the Vitek^®^ system faces certain limitations, including the significant manual effort required, a prolonged turnaround time, and an inability to identify several key bacterial pathogens relevant to the aquaculture industry. Moreover, Haung et al. [40] clarified that *A. dhakensis* was primarily misidentified as *A. hydrophila* via the VITEK-2 system, while its identity was confirmed by sequencing. Herein, the 16S rRNA partial gene sequence of recovered isolates were identical to those of their related species on GenBank database, with a similarity 100%, then has been accepted and published on GenBank under accession number PV018986. El Latif et al. [41] elucidated that 16S rRNA partial gene sequencing and phylogenetic analysis succeeded to verify the identity of *A. veronii* isolates.

*S. agalactiae* was the most predominant isolate among others during summer 2023 and 2024. This bacterium has emerged as a growing concern in aquaculture worldwide affecting a variety of fish species particularly those raised in warm water [42]. It causes streptococcosis, which can lead to high morbidity and mortality rates among the infected fish [43]. *S. agalactiae* has been frequently encountered in unusual mass mortality events that are reported among farmed tilapia during summer. These findings support the idea that summer outbreaks clarified by Abu-Elala et al. [16] in Kafr Elsheikh province and by Abdallah et al. [12] in Assiut province. Another survey confirmed that *S. agalactiae* was the third most prevalent pathogen following *Aeromonas* and *Vibrio* species causing massive mortalities of cultured tilapia during the summer months at Beheira, Fayoum, Ismailia, Kafr El Sheikh, and Sharqia provinces [14]. Nervous signs or erratic swimming, skin darkness, “pop eye” with hemorrhages and corneal opacity were the most common signs associated with *S. agalactiae* infection [44]. The *cylE* gene is highlighted as a significant virulence factor, contributing to the development of characteristic signs and lesions [45]. Furthermore, the detection of this gene within *S. agalactiae* isolates in Nile tilapia confirms its involvement in the pathogenesis of these streptococcal infections [46].

*Vibrio* sp. ranked the second most prevalent pathogen with a prevalence rate of 34.9% during autumn season. Vibriosis is primarily associated with brackish and marine aquaculture, however, the ability of *Vibrio* strains to infect farmed tilapia in freshwater and low-salinity environments has also been recorded [47, 48]. Our results resemble the previous study that investigated mortality outbreak in cultured tilapia caused by *V. mimicus* and *V. cholerae* during autumn season at Kafr El-Sheikh province [49]. Further study reported a case of co-infection between *V. alginolyticus*, *Aeromonas* sp., and *E. faecalis* in poly-cultured Nile tilapia and African catfish during early autumn at Port Said governorate [50]. *V. alginolyticus* and *V. vulnificus* were also identified in cultured *O. niloticus* in Al Fayoum governorate [51]. A study was conducted by Elgohary et al. [19] confirmed that the highest prevalence of *V. vulnificus* in autumn season is within tilapia farms.

*V. campbellii* and *V. owensii* were often related to acute hepatopancreatic necrosis disease affecting early phases of penaeid shrimp, leading to a decrease in their survival rates [52, 53]. However, a study investigated *V. campbellii* for the first time as a newly emerging pathogen affecting Egyptian farmed gilthead seabream [54]. The present work represents the first study to identify *V. campbellii* and *V. owensii* in cultured tilapia farms. This may be attributed to *Vibrio* species are characterized by high genome plasticity due to recombination, frequent mutation, and lateral gene transfer allowing *Vibrio* to adapt rapidly to environmental alterations [55]. Several studies highlighted the relationship between water quality parameters and epidemiology of *Vibrio* strains. Winfield [56] established a strong correlation between the incidence of *Vibrio* outbreaks and temperature fluctuations, particularly, in spring and fall seasons. Moreover, poor water quality measures and bad managemental practices exacerbated the *Vibrio* infection in tilapia, especially cultured in earthen ponds [21]. Anorexia, lethargy, red spots, dark skin, corneal opacity, exophthalmia, congestion in hematopoietic tissues as well as distended intestines were the typical *Vibrio* signs in infected fish [57]. Their ability to cause disease is related to a variety of virulence factors, especially hemolysins [58]. The presence of *trh*, *tlh*, and *tdh* genes in *Vibrio* spp. refers to their pathogenicity that might correspond with the potential of infections and diseases [59, 60].

*A. veronii* was the third most widespread bacterial pathogens isolated from farmed tilapia during autumn season. Disease and mortality caused by motile aeromonads in freshwater fish have been mainly linked with *A. hydrophila* whereas other species were probably ignored. Nonetheless, it has been attributed to various virulent *Aeromonas* sp., including *A. veronii*, *A. caviae*, *A. sobria*, *A. jandaei* and *A. hydrophila* [61–64]. *A. veronii* is ubiquitous in the aquatic environment that are often termed as fish pathogen causing epizootic ulcerative syndrome and hemorrhagic septicemia [65]. Egyptian fish farms have experienced frequent outbreaks of *A. veronii* infection during the summer season among farmed tilapia within different localities [41, 66, 67]. In accordance with the present findings, previous study has demonstrated that *A. veronii* can multiply faster and cause serious infections under adverse conditions during both summer and autumn seasons [68]. Ulcerations over the body surface, hemorrhage, and congestion in internal organs affecting the liver, kidney, and spleen were specific signs of Aeromonad infection [69]. Extracellular hydrolytic enzymes such as hemolysins, proteases, aerolysin, phospholipases, and cytotoxic enterotoxins might contribute to their disease progression in fish [2, 70].

*E. faecalis* has also been identified to cause hemorrhagic septicemia in Nile tilapia, with high mortality rates during late autumn. Repeated outbreaks in aquaculture worldwide, including Egypt, have been associated with *E. faecalis* [71–74]. Tilapia were highly susceptible to *E. faecalis* infection owing to sewage pollution in Egyptian fish farms or poultry manure which used as a natural fertilizer in ponds [75, 76]. Our findings agreed with those surveyed the natural outbreaks of *E. faecalis* infection in *O. niloticus* with the highest infection rate in both autumn and summer seasons at El Fayoum and the highest prevalence was recorded in the winter season at El Sharkia province [17]. It has been characterized to cause septicemic signs in tilapia fish such as skin hemorrhage, ulcers, dark discoloration, exophthalmia, eye opacity, abdominal ascites, pale or hemorrhagic liver, enlarged spleen, hemorrhagic kidney, and brain [77]. One of the important virulence determinants of *E. faecalis* is gelatinase toxin that hydrolyzes gelatin, hemoglobin, and collagen which is essential for the bacteria to cause disease [78, 79].

Stress is considered the major factor of the fish susceptibility to diseases [80]. The identified bacterial strains could be natural inhabitants of aquatic environment such as *Vibrio* and *Aeromonas* sp. or introduced as new pathogens for tilapia aquaculture such as *Enterococcus* and *Streptococcus* sp [18, 74, 81, 82]. Contaminated agriculture drainage water and untreated poultry manure used as organic fertilizer have participated in adding more pathogens to fish farms [83]. Most fish diseases caused by bacteria stem from secondary invaders targeting hosts that are already immune-compromised or subjected to environmental stress [84]. Poor water parameters, including a surplus of organic material or ammonia, elevated heat, and reduced oxygen levels, provide an optimal setting for these pathogens to thrive [85]. Adverse management practices such as handling or crowding, lack of biosecurity, intensive culture in the affected farms are potent risk factors impairing the defense mechanisms of tilapia fish and increasing the eruption and severity of those infections [39].

Regarding the antibiogram, the isolated strains showed a high degree of resistance to antibiotics most frequently used and prescribed in Egypt. All isolates exhibited complete resistance to penicillin, ampicillin, novobiocin, and trimethoprim/sulfamethoxazole. A remarkable resistance against kanamycin, erythromycin, and amoxycillin/clavulanic acid was detected in *Vibrio* sp. These results match those observed in Ayoub et al. [86], who explained that high antimicrobial resistance against ampicillin, amoxicillin, and erythromycin expressed by *Vibrio*, *Aeromonas*, and *Pseudomonas* sp. which recovered from diseased Nile tilapia. In the study, moderate antibiotic susceptibility was also found in *V. alginolyticus* to ciprofloxacin and *V. campbellii* to oxytetracycline. Moreover, *A. veronii* was found to be highly resistant to all tested antibiotics. Elgendy et al. [21] identified *A. veronii* in Egypt with antimicrobial resistance to ampicillin, amoxicillin, gentamicin, and neomycin. However, those authors clarified that all isolates were sensitive to ciprofloxacin, one isolate was sensitive to oxytetracycline, and one isolate showed moderate susceptibility to sulfamethoxazole/trimethoprim. The present findings seem to be consistent with dos Santos et al. [87], who investigated *A. veronii* in Brazil showing resistance to three antimicrobials: enrofloxacin, oxytetracycline, and amoxicillin, while the susceptibility only to florfenicol.

The antimicrobial sensitivity profile of *S. agalactiae* exhibited variable resistance to antibiotics. It expressed total resistance to erythromycin, and kanamycin, while the bacterial isolate was intermediately susceptible to ciprofloxacin, oxytetracycline, and amoxycillin/clavulanic acid. These findings agree with Abu-Elala et al. [16], who illustrated that *Streptococcus*, *Enterococcus*, and *Lactococcus* sp. displayed significant resistance to ampicillin, amoxicillin, oxytetracycline, tetracycline, and neomycin. However, these bacterial isolates showed high susceptibility to ciprofloxacin, gentamycin, and trimethoprim/sulfamethoxazole. Osman et al. [18] also identified *Streptococcus* spp. with abundant antibiotic resistance to ampicillin, penicillin, erythromycin, chloramphenicol, rifampicin, vancomycin, clindamycin, ofloxacin, and tetracycline. The antimicrobial sensitivity patterns in this study were only detected in *E. faecalis* strain to ciprofloxacin, whereas it displayed moderate susceptibility to oxytetracycline, erythromycin, and kanamycin. Hence, ciprofloxacin is effective against this bacterium and can be a drug of choice to treat affected fish. This also accords with Osman et al. [88], who demonstrated that all *E. faecalis* isolates were susceptible for penicillin, gentamicin, nitrofurantoin, and streptomycin. As well, most of the isolates were susceptible to ciprofloxacin, ampicillin, chloramphenicol, and vancomycin. In our study, *E. faecalis* was resistant only to amoxycillin/clavulanic acid. Arumugam et al. [89] confirmed that this isolate showed resistance to amoxiclav, oxytetracycline, ampicillin, kanamycin, erythromycin, gentamicin, nitrofurantoin, streptomycin, penicillin-G, and sulphafurazole and was intermediately resistant to ciprofloxacin, chloramphenicol, norfloxacin, clindamycin, and vancomycin.

The uncontrolled and indiscriminate application of antibiotics in fish farming is a major contributor to the emergence of drug-resistant bacteria [90, 91]. In Egypt, some antimicrobials usually prescribed without veterinarian to be use in aquaculture [92]. Some of those agents are used prophylactically in fish farming either to promote growth or to prevent the risk of disease incidences [93]. The long-term or misuse of these chemicals not only leaves residues but leads to the emergence of resistant bacteria [94]. The worrying findings of antimicrobial susceptibility analysis emphasize restricting the antibiotics used in aquaculture by developing drug dosage guides for antimicrobial treatment of diseased fish. Thus, it is urgently necessary to outlaw the preventative application of antibiotics in raising fish [95, 96].

## Conclusion

In short, *V. alginolyticus*, *V. campbellii*, *V. owensii*, *A. veronii*, *E. faecalis*, and *S. agalactiae* were identified from heavy mortalities of pond-farmed Nile tilapia. These bacteria were isolated from fish with hemorrhagic septicemia and mortality cases among cultured *O. niloticus* in Beheira province. The findings highlighted that *S. agalactiae* was the most prevalent one during high temperatures of the summer season. *V. campbellii* and *V. owensii* have emerged as new candidate bacterial strains for tilapia aquaculture. The most concerning aspect is the existence of high resistance among the strains to most of the tested antimicrobial discs particularly, novobiocin, trimethoprim/sulfamethoxazole, penicillin, and ampicillin. Therefore, biosecurity plans and improving farming practices could be a better choice to reduce the occurrence of such infections in the affected farms. Controlling the haphazard use of antimicrobials is also crucial. Further research and monitoring should be implemented to develop effective treatment and vaccination for controlling bacterial infections as well as enhancing the sustainability and health of tilapia farming.

## Supplementary Information

Supplementary Material 1.

## Data Availability

The datasets used and/or analyzed during the current study available from the corresponding author on reasonable request.

## References

[1] Hassan MMA, Khalil RH, Abotaleb MM, Amer MT, Abdel-Latif HMR. Bacterial pathogens and their antimicrobial resistance in farmed nile tilapia experiencing summer mortality in Kafr El-Sheikh, Egypt. In: Microorganisms. 2025;13. 10.3390/microorganisms13112448 PMC12654396 41304134

[2] Abdel-Latif HMR, Khafaga AF. Natural co-infection of cultured nile tilapia Oreochromis niloticus with Aeromonas hydrophila and gyrodactylus cichlidarum experiencing high mortality during summer. Aquac Res. 2020;51(5):1880–92.

[3] Bentzon-Tilia M, Sonnenschein EC, Gram L. Monitoring and managing microbes in aquaculture – Towards a sustainable industry. Microb Biotechnol. 2016;9(5):576–84. 27452663 10.1111/1751-7915.12392 PMC4993175

[4] Elgendy MY, Awad ES, Darwish DA, Ibrahim TB, Soliman WSE, Kenawy AM, Abumourad IMK, Abbas HH, Abbas WT. Investigations on the influence of Moringa Oleifera on the growth, haematology, immunity and disease resistance in Oreochromis niloticus with special reference to the analysis of antioxidant activities by PAGE electrophoresis. Aquac Res. 2021;52(10):4983–95.

[5] Balta F, Dengiz Balta Z. Determination of antimicrobial activity and MIC value of Tannic acid against four different fish pathogens. J Anatol Environ Anim Sci. 2024;9(4):582–9.

[6] Ali SE, Jansen MD, Mohan CV, Delamare-Deboutteville J, Charo-Karisa H. Key risk factors, farming practices and economic losses associated with tilapia mortality in Egypt. Aquaculture. 2020;527:735438.

[7] Haenen OLM, Dong HT, Hoai TD, Crumlish M, Karunasagar I, Barkham T, Chen SL, Zadoks R, Kiermeier A, Wang B, et al. Bacterial diseases of tilapia, their zoonotic potential and risk of antimicrobial resistance. Reviews Aquaculture. 2023;15(S1):154–85.

[8] Zaheen Z, War AF, Ali S, Yatoo AM, Ali MN, Ahmad SB, Rehman MU, Paray BA. Chap. 7 - Common bacterial infections affecting freshwater fish fauna and impact of pollution and water quality characteristics on bacterial pathogenicity. In: *Bacterial Fish Diseases.* edn. Edited by Dar GH, Bhat RA, Qadri H, Al-Ghamdy KM, Hakeem KR: Academic Press. 2022:133–154.

[9] Abu-Elala NM, Abd‐Elsalam RM, Marouf S, Abdelaziz M, Moustafa M. Eutrophication, ammonia Intoxication, and infectious diseases: interdisciplinary factors of mass mortalities in cultured nile tilapia. J Aquat Anim Health. 2016;28(3):187–98. 27484819 10.1080/08997659.2016.1185050

[10] El-Son MAM, Nofal MI, Abdel-Latif HMR. Co-infection of Aeromonas hydrophila and vibrio parahaemolyticus isolated from diseased farmed striped mullet (Mugil cephalus) in Manzala, Egypt – A case report. Aquaculture. 2021;530:735738.

[11] Sarkar P, Issac PK, Raju SV, Elumalai P, Arshad A, Arockiaraj J. Pathogenic bacterial toxins and virulence influences in cultivable fish. Aquac Res. 2021;52(6):2361–76.

[12] Abdallah ESH, Metwally WGM, Bayoumi SALH, Abdel Rahman MAM, Mahmoud MM. Isolation and characterization of Streptococcus agalactiae inducing mass mortalities in cultured nile tilapia (Oreochromis niloticus) with trials for disease control using zinc oxide nanoparticles and ethanolic leaf extracts of some medicinal plants. BMC Vet Res. 2024;20(1):468. 39402574 10.1186/s12917-024-04298-z PMC11475875

[13] Huicab-Pech ZG, Castaneda-Chavez MR, Lango-Reynoso F. Pathogenic bacteria in Oreochromis niloticus Var. Stirling tilapia culture. Fisheries Aquaculture J. 2017;8(2):1–7.

[14] Ali SE, Mahana O, Mohan CV, Delamare-Deboutteville J, Elgendy MY. Genetic characterization and antimicrobial profiling of bacterial isolates collected from nile tilapia (Oreochromis niloticus) affected by summer mortality syndrome. J Fish Dis. 2022;45(12):1857–71. 36057979 10.1111/jfd.13710

[15] Magouz FI, Moustafa EM, Abo-Remela EM, Halawa MR, Barakaat PM, Omar AA. Summer mortality syndrome bacterial pathogens in farmed nile tilapia (Oreochromis niloticus). Open Veterinary J. 2024;14(1):53–69. 10.5455/OVJ.2024.v14.i1.7 PMC11018447 38633195

[16] Abu-Elala NM, Abd-Elsalam RM, Younis NA. Streptococcosis, lactococcosis and enterococcosis are potential threats facing cultured nile tilapia (Oreochomis niloticus) production. Aquac Res. 2020;51(10):4183–95.

[17] Elgohary I, Eissa AE, Fadel NG, Ibrahim Abd Elatief J, Mahmoud MA. Bacteriological, molecular, and pathological studies on the Gram-positive bacteria aerococcus viridans and Enterococcus faecalis and their effects on Oreochromis niloticus in Egyptian fish farms. Aquac Res. 2021;52(5):2220–32.

[18] Osman KM, Al-Maary KS, Mubarak AS, Dawoud TM, Moussa IMI, Ibrahim MDS, Hessain AM, Orabi A, Fawzy NM. Characterization and susceptibility of Streptococci and enterococci isolated from nile tilapia (Oreochromis niloticus) showing septicaemia in aquaculture and wild sites in Egypt. BMC Vet Res. 2017;13(1):357. 29178882 10.1186/s12917-017-1289-8 PMC5702248

[19] Elgohary I, Abd Elatief I, Fadel JG, Eissa A NE, Mahmoud A. Pathological, bacteriological and seasonal prevalence of Aeromonas hydrophila, vibrio vulnificus, proteus vulgaris and Pseudomonas aeruginosa; infecting Oreochromis niloticus in some Egyptian fish farms. Egypt J Aquat Biology Fisheries. 2020;24(5):467–82.

[20] Balta F, Sandalli C, Kayis S, Ozgumus O. Molecular analysis of antimicrobial resistance in yersinia ruckeri strains isolated from rainbow trout (Oncorhynchus mykiss) grown in commercial fish farms in Turkey. Bull Eur Association Fish Pathologists. 2010;30(6):211–9.

[21] Elgendy MY, Shaalan M, Abdelsalam M, Eissa AE, El-Adawy MM, Seida AA. Antibacterial activity of silver nanoparticles against antibiotic-resistant Aeromonas veronii infections in nile tilapia, Oreochromis niloticus (L.), in vitro and in vivo assay. Aquac Res. 2022;53(3):901–20.

[22] Santos L, Ramos F. Antimicrobial resistance in aquaculture: current knowledge and alternatives to tackle the problem. Int J Antimicrob Agents. 2018;52(2):135–43. 29567094 10.1016/j.ijantimicag.2018.03.010

[23] Austin B, Austin DA. Characteristics of the pathogens: Gram-negative bacteria. In: Bacterial Fish Pathogens: Diseases of Farmed and Wild Fish. edn. Edited by Austin B, Austin DA. Dordrecht: Springer Netherlands. 2007:81–150.

[24] Buller NB. Bacteria and fungi from fish and other aquatic animals: a practical identification manual 2nd Edition: Cabi; 2014.

[25] Sultana T, Siddique AB, Akther S, Ahmed S, Shahadat MN, Billah MB, Rahman MH. Prevalence and antibiotic resistance patterns of vibrio cholerae and vibrio parahaemolyticus isolated from common fish of retail markets in Dhaka, Bangladesh. Discover Bacteria. 2025;2(1):23.

[26] Tarr Cheryl L, Patel Jayna S, Puhr Nancy D, Sowers Evangeline G, Bopp Cheryl A, Strockbine Nancy A. Identification of vibrio isolates by a multiplex PCR assay and RpoB sequence determination. J Clin Microbiol. 2007;45(1):134–40. 17093013 10.1128/JCM.01544-06 PMC1828960

[27] Gordon L, Giraud E, Ganière JP, Armand F, Bouju-Albert A, De La Cotte N, Mangion C, Le Bris H. Antimicrobial resistance survey in a river receiving effluents from freshwater fish farms. J Appl Microbiol. 2007;102(4):1167–76. 17381761 10.1111/j.1365-2672.2006.03138.x

[28] Lagacé L, Pitre M, Jacques M, Roy D. Identification of the bacterial community of maple Sap by using amplified ribosomal DNA (rDNA) restriction analysis and rDNA sequencing. Appl Environ Microbiol. 2004;70(4):2052–60. 15066796 10.1128/AEM.70.4.2052-2060.2004 PMC383098

[29] Mashouf RY, Mousavi SM, Rabiee S, Alikhani MY, Arabestani MR. Direct identification of *Streptococcus agalactiae* in vaginal colonization in pregnant women using polymerase chain reaction. J Compr Pediatr. 2014;5(4):e23339.

[30] Kumar S, Stecher G, Suleski M, Sanderford M, Sharma S, Tamura K. MEGA12: molecular evolutionary genetic analysis version 12 for adaptive and green computing. Mol Biol Evol. 2024;41(12):msae263. 39708372 10.1093/molbev/msae263 PMC11683415

[31] CLSI. Performance standards for antimicrobial susceptibility Testing; clinical and laboratory standards Institute.Twenty-Fourth informational Supplement. In.: CLSI Wayne. PA; 2014.

[32] Jantrakajorn S, Maisak H, Wongtavatchai J. Comprehensive investigation of streptococcosis outbreaks in cultured nile Tilapia, Oreochromis niloticus, and red Tilapia, Oreochromis sp., of Thailand. J World Aquaculture Soc. 2014;45(4):392–402.

[33] Korni FMM, EL-Nahass E-S, Ahmed WMS. An outbreak of motile Aeromonas septicemia in cultured nile tilapia, Oreochromis niloticus with reference to hematological, biochemical and histopathological alterations. J Fish Pathol. 2017;30(1):11–24.

[34] Ador MAA, Haque MS, Paul SI, Chakma J, Ehsan R, Rahman AJAS. Potential application of PCR based molecular methods in fish pathogen identification: a review. Aquaculture Stud. 2021;22(1). AQUAST621.

[35] Küpfer M, Kuhnert P, Korczak BM, Peduzzi R, Demarta A. Genetic relationships of Aeromonas strains inferred from 16S rRNA, GyrB and RpoB gene sequences. Microbiol Soc. 2006;56(12):2743–51. 10.1099/ijs.0.63650-0 17158971

[36] Lal D, Verma M, Lal R. Exploring internal features of 16S rRNA gene for identification of clinically relevant species of the genus Streptococcus. Ann Clin Microbiol Antimicrob. 2011;10(1):28. 21702978 10.1186/1476-0711-10-28 PMC3151204

[37] Ryu H, Henson M, Elk M, Toledo-Hernandez C, Griffith J, Blackwood D, Noble R, Gourmelon M, Glassmeyer S, Santo Domingo Jorge W. Development of quantitative PCR assays targeting the 16S rRNA genes of Enterococcus spp. And their application to the identification of Enterococcus species in environmental samples. Appl Environ Microbiol. 2013;79(1):196–204. 23087032 10.1128/AEM.02802-12 PMC3536114

[38] Yong L, Guanpin Y, Hualei W, Jixiang C, Xianming S, Guiwei Z, Qiwei W, Xiuqin S. Design of vibrio 16S rRNA gene specific primers and their application in the analysis of seawater vibrio community. J Ocean Univ China. 2006;5(2):157–64.

[39] Abdelsalam M, Elgendy MY, Elfadadny MR, Ali SS, Sherif AH, Abolghait SK. A review of molecular diagnoses of bacterial fish diseases. Aquacult Int. 2023;31(1):417–34.

[40] Huang M, Chen H, Li C, Liu Y, Gan C, El-Sayed Ahmed MAE-G, Liu R, Shen C, Zhong R, Tian G-B. Rapid fulminant progression and mortality secondary to Aeromonas Dhakensis septicemia with hepatitis B virus infection following the ingestion of Snakehead fish in Mainland china: a case report. Foodborne Pathog Dis. 2020;17(12):743–9. 32985901 10.1089/fpd.2019.2780

[41] El Latif AMA, Elabd H, Amin A, Eldeen AIN, Shaheen AA. High mortalities caused by Aeromonas veronii:identification, pathogenicity, and histopathologicalstudies in Oreochromis niloticus. Aquacult Int. 2019;27(6):1725–37.

[42] Geng Y, Wang KY, Huang XL, Chen DF, Li CW, Ren SY, Liao YT, Zhou ZY, Liu QF, Du ZJ, et al. Streptococcus agalactiae, an emerging pathogen for cultured Ya-Fish, schizothorax prenanti, in China. Transbound Emerg Dis. 2012;59(4):369–75. 22146014 10.1111/j.1865-1682.2011.01280.x

[43] Amal MNA, Saad MZ, Zahrah AS, Zulkafli AR. Water quality influences the presence of Streptococcus agalactiae in cage cultured red hybrid tilapia, Oreochromis niloticus × Oreochromis mossambicus. Aquac Res. 2015;46(2):313–23.

[44] Mishra A, Nam G-H, Gim J-A, Lee H-E, Jo A, Kim H-S. Current challenges of Streptococcus infection and effective Molecular, Cellular, and environmental control methods in aquaculture. Mol Cells. 2018;41(6):495–505. 29754470 10.14348/molcells.2018.2154 PMC6030242

[45] Kannika K, Pisuttharachai D, Srisapoome P, Wongtavatchai J, Kondo H, Hirono I, Unajak S, Areechon N. Molecular serotyping, virulence gene profiling and pathogenicity of Streptococcus agalactiae isolated from tilapia farms in Thailand by multiplex PCR. J Appl Microbiol. 2017;122(6):1497–507. 28295891 10.1111/jam.13447

[46] Algammal AM, Mabrok M, Almessiry BK, Atwah B, Al-otaibi AS, Mohamed YS, Steele S, Enany M, Dayrit GB, Yousseff FM, et al. Unraveling the pathogenic potential, virulence traits, and antibiotic resistance genes of multidrug-resistant Streptococcus agalactiae strains retrieved from nile tilapia. BMC Microbiol. 2025;25(1):629. 41044671 10.1186/s12866-025-04405-6 PMC12492693

[47] Chen C-Y, Chao C-B, Bowser PR. Infection of tilapia Oreochromis sp. by vibrio vulnificus in freshwater and Low-salinity environments. J World Aquaculture Soc. 2006;37(1):82–8.

[48] Sony M, Sumithra TG, Anusree VN, Amala PV, Reshma KJ, Alex S, Sanil NK. Antimicrobial resistance and virulence characteristics of vibrio vulnificus, vibrio parahaemolyticus and vibrio harveyi from natural disease outbreaks of marine/estuarine fishes. Aquaculture. 2021;539:736608.

[49] Elgendy MY, Abdelsalam M, Kenawy AM, Ali SE. Vibriosis outbreaks in farmed nile tilapia (Oreochromis niloticus) caused by vibrio mimicus and V. cholerae. Aquacult Int. 2022;30(5):2661–77.

[50] Abdelsalam M, Ewiss MAZ, Khalefa HS, Mahmoud MA, Elgendy MY, Abdel-Moneam DA. Coinfections of Aeromonas spp., Enterococcus faecalis, and vibrio alginolyticus isolated from farmed nile tilapia and African catfish in Egypt, with an emphasis on poor water quality. Microb Pathog. 2021;160:105213. 34582943 10.1016/j.micpath.2021.105213

[51] Younes A, Fares M, Gaafar A, Mohamed L. Isolation of vibrio alginolyticus and vibrio vulnificus strains from cultured Oreochromis niloticus around Qarun Lake, Egypt. Global Vet. 2016;16:01–5.

[52] Liu L, Xiao J, Zhang M, Zhu W, Xia X, Dai X, Pan Y, Yan S, Wang Y. A vibrio Owensii strain as the causative agent of AHPND in cultured shrimp, Litopenaeus vannamei. J Invertebr Pathol. 2018;153:156–64. 29427635 10.1016/j.jip.2018.02.005

[53] Srisangthong I, Sangseedum C, Chaichanit N, Surachat K, Suanyuk N. Mittraparp-arthorn P: characterization and genome analysis of vibrio Campbellii lytic bacteriophage OPA17. Microbiol Spectr. 2023;11(2):e01623–01622. 36719217 10.1128/spectrum.01623-22 PMC10101143

[54] Aly SM, Elatta MA, ElBanna NI, El-Shiekh MA, Kelany MS, Fathi M, Mabrok M. Studies on vibrio Campbellii as a newly emerging pathogen affecting cultured seabream (Sparus aurata) in Egypt. Aquacult Int. 2024;32(2):1685–701.

[55] Oberbeckmann S, Fuchs BM, Meiners M, Wichels A, Wiltshire KH, Gerdts G. Seasonal dynamics and modeling of a vibrio community in coastal waters of the North sea. Microb Ecol. 2012;63(3):543–51. 22202887 10.1007/s00248-011-9990-9

[56] Winfield IJ, SEA BASS AND SEA BREAM: A PRACTICAL APPROACH TO DISEASE CONTROL AND HEALTH MANAGEMENT. J Fish Biol. 2018;93(2):434–434.

[57] Frans I, Michiels CW, Bossier P, Willems KA, Lievens B, Rediers H. Vibrio anguillarum as a fish pathogen: virulence factors, diagnosis and prevention. J Fish Dis. 2011;34(9):643–61. 21838709 10.1111/j.1365-2761.2011.01279.x

[58] Darshanee Ruwandeepika HA, Sanjeewa Prasad Jayaweera T, Paban Bhowmick P, Karunasagar I, Bossier P, Defoirdt T. Pathogenesis, virulence factors and virulence regulation of vibrios belonging to the harveyi clade. Reviews Aquaculture. 2012;4(2):59–74.

[59] Algammal AM, Mabrok M, Alfifi KJ, Alghamdi S, Alammari DM, Ghobashy MOI, Alshahrani MY, El-Demerdash AS, Eissa E-SH, Elalamy AM, et al. The evolving multidrug-resistant V. alginolyticus in sea Bream commonly harbored collagenase, trh, and Tlh virulence genes and sul1, blaTEM, aadA, tetA, blaOXA, and TetB or TetM resistance genes. Aquacult Int. 2025;33(2):134.

[60] Paria P, Behera BK, Mohapatra PKD, Parida PK. Virulence factor genes and comparative pathogenicity study of tdh, Trh and Tlh positive vibrio parahaemolyticus strains isolated from whiteleg shrimp, Litopenaeus vannamei (Boone, 1931) in India. Infect Genet Evol. 2021;95:105083. 34536578 10.1016/j.meegid.2021.105083

[61] Abd-Elall A, Abd-El-Kader M, Atia AS. Occurrence, seasonal variations and virulence of Aeromonas hydrophila and Aeromonas caviae in fish farms at East Delta, Egypt. Global Vet. 2014;13(3):328–36.

[62] Anantasuk N, Phurahong T, Pumchan A, Hirono I, Unajak S. Molecular characterization and bivalent vaccine development of Aeromonas hydrophila and Aeromonas veronii in nile tilapia (Oreochromis niloticus). Aquaculture. 2024;590:741042.

[63] Dar GH, Dar SA, Kamili AN, Chishti MZ, Ahmad F. Detection and characterization of potentially pathogenic Aeromonas sobria isolated from fish hypophthalmichthys molitrix (Cypriniformes: Cyprinidae). Microb Pathog. 2016;91:136–40. 26518124 10.1016/j.micpath.2015.10.017

[64] Dong HT, Techatanakitarnan C, Jindakittikul P, Thaiprayoon A, Taengphu S, Charoensapsri W, Khunrae P, Rattanarojpong T, Senapin S. Aeromonas jandaei and Aeromonas veronii caused disease and mortality in nile tilapia, Oreochromis niloticus (L). J Fish Dis. 2017;40(10):1395–403. 28383126 10.1111/jfd.12617

[65] Aly SM, Abou-El-Atta ME, El-Mahallawy HS, Elaswad A, ElAbyad FA, ElBanna NI. Aeromonas veronii and ulcerative syndrome in cultured nile tilapia (Oreochromis niloticus) and their associated factors. Aquacult Int. 2023;31(5):2867–81.

[66] Fadel A, Mahmoud MA, Abdelsalam M, Eissa E-SH, Sherif AH. Aeromonas veronii infection in cultured Oreochromis niloticus: prevalence, molecular and histopathological characterization correlated to water physicochemical characteristics, with the protective autochthonous probiotic. Aquacult Int. 2025;33(4):298.

[67] Reda RM, El-Murr A, Abd Elhakim Y, El-Shahat W. Aeromonas veronii detection in Egyptian fish farms with summer tilapia mortality outbreaks and the role of formic acid in limiting its spread. Aquac Res. 2022;53(3):940–56.

[68] Chen C, Zu S, Zhang D, Zhao Z, Ji Y, Xi H, Shan X, Qian A, Han W, Gu J. Oral vaccination with Recombinant Lactobacillus casei expressing Aha1 fused with CTB as an adjuvant against Aeromonas veronii in common carp (Cyprinus carpio). Microb Cell Fact. 2022;21(1):114. 35698139 10.1186/s12934-022-01839-9 PMC9191526

[69] Wang B, Mao C, Feng J, Li Y, Hu J, Jiang B, et al. A first report of Aeromonas veronii infection of the sea Bass, lateolabrax maculatus in China. Front Veterinary Sci. 2021;7–2020. 10.3389/fvets.2020.600587. 10.3389/fvets.2020.600587 PMC7855973 33553279

[70] Beaz-Hidalgo R, Figueras MJ. Aeromonas spp. Whole genomes and virulence factors implicated in fish disease. J Fish Dis. 2013;36(4):371–88. 23305319 10.1111/jfd.12025

[71] Ezzat M, Mabrok M, Abd El Moez NT, Abdullah O, Wahdan A. Prevalence and resistance pattern of Enterococcus faecalis in cultured Oreochromis niloticus at Ismailia Governorate, Egypt. Suez Canal Veterinary Med J. 2024;29(2):269–79.

[72] Hassan MA, Abdel-Naeim NS, Mabrok M, Dessouki AA, Hassan AM. Isolation and identification of Enterococcus faecalis from cultured Oreochromis niloticus and Mugil cephalus with a special emphasis on a possible integrated control strategy. Aquac Res. 2022;53(16):5521–35.

[73] Rahman M, Rahman MM, Deb SC, Alam MS, Alam MJ, Islam MT. Molecular identification of multiple antibiotic resistant fish pathogenic Enterococcus faecalis and their control by medicinal herbs. Sci Rep. 2017;7(1):3747. 28623336 10.1038/s41598-017-03673-1 PMC5473830

[74] Kumru S, Balta ZD, Aliu H, Balta F. Identification of Enterococcus species isolated from commercial fish feeds and infected fish specimens. J Hellenic Veterinary Med Soc. 2024;75(2):7523–30.

[75] Shaker IM, Abou Zead MY, Farouk AED. Economic impacts of ponds management on water quality and growth performance of fish in polyculture. Egypt J Aquat Biology Fisheries. 2016;20(4):47–60.

[76] Zahran E, Mahgoub HA, Abdelhamid F, Sadeyen J-R, Risha E. Experimental pathogenesis and host immune responses of Enterococcus faecalis infection in nile tilapia (Oreochromis niloticus). Aquaculture. 2019;512:734319.

[77] El-Nobi GA, Hassanin M, El-Hady M, Aboshabana S. Isolation, identification, molecular, and histopathological investigations of two pathogenic Enterococcus species from tilapia in Egyptian farms. Slovenian Veterinary Research/Slovenski Veterinarski Zbornik. 2021;58.

[78] Aboyadak I, Ali NG. Enrofloxacin, effective treatment of Pseudomonas aeruginosa and Enterococcus faecalis infection in Oreochromis niloticus. In: Microorganisms. 2024;12. 10.3390/microorganisms12050901 PMC11124035 38792731

[79] Akter T, Haque MN, Ehsan R, Paul SI, Foysal MJ, Tay ACY, Islam MT, Rahman MM. Virulence and antibiotic-resistance genes in Enterococcus faecalis associated with streptococcosis disease in fish. Sci Rep. 2023;13(1):1551. 36707682 10.1038/s41598-022-25968-8 PMC9883459

[80] Winton JR. Fish health management, 2nd edition. American Fisheries Society,Bethesda, Maryland. 2001:559–640.

[81] Brumfield KD, Usmani M, Chen KM, Gangwar M, Jutla AS, Huq A, Colwell RR. Environmental parameters associated with incidence and transmission of pathogenic vibrio spp. Environ Microbiol. 2021;23(12):7314–40. 34390611 10.1111/1462-2920.15716

[82] Huddleston Jennifer R, Zak John C, Jeter Randall M. Antimicrobial susceptibilities of Aeromonas spp. Isolated from environmental sources. Appl Environ Microbiol. 2006;72(11):7036–42. 16950901 10.1128/AEM.00774-06 PMC1636150

[83] Badawy TEM, Zaki EM, Kenawy DA. Risk assessment of using poultry manure on water quality, fish as well as their human health and biological treatment in aquaculture ponds. In.: Massive Conferences and Trade Fairs. 2009:1–22.

[84] Derome N, Gauthier J, Boutin S, Llewellyn M. Bacterial Opportunistic Pathogens of Fish. In: The Rasputin Effect: When Commensals and Symbionts Become Parasitic. edn. Edited by Hurst CJ. Cham: Springer International Publishing. 2016:81–108.

[85] Abd El-Hack ME, El-Saadony MT, Nader MM, Salem HM, El-Tahan AM, Soliman SM, Khafaga AF. Effect of environmental factors on growth performance of nile tilapia (Oreochromis niloticus). Int J Biometeorol. 2022;66(11):2183–94. 36044083 10.1007/s00484-022-02347-6 PMC9640449

[86] Ayoub HF, Tohamy EY, Mohamed SS. Isolation, identification And antimicrobial profile of Aeromonas spp., Pseudomonas spp. And vibrio spp. From the nile Tilapia, Oreochromis niloticus in fish farms. Egypt J Aquat Biology Fisheries. 2021;25(3):171.

[87] dos Santos SB, Alarcon MF, Ballaben AS, Harakava R, Galetti R, Guimarães MC, Natori MM, Takahashi LS, Ildefonso R, Rozas-Serri M. First report of Aeromonas veronii as an emerging bacterial pathogen of farmed nile tilapia (Oreochromis niloticus) in Brazil. In: Pathogens 12; 2023. 10.3390/pathogens12081020 PMC10459805 37623980

[88] Osman KM, Ali MN, Radwan I, ElHofy F, Abed AH, Orabi A, et al. Dispersion of the Vancomycin resistance genes VanA and VanC of Enterococcus isolated from nile tilapia on retail sale: a public health hazard. Front Microbiol. 2016;7–2016. 10.3389/fmicb.2016.01354. 10.3389/fmicb.2016.01354 PMC4999479 27617012

[89] Arumugam U, Stalin N, Rebecca GP. Isolation, molecular identification and antibiotic resistance of Enterococcus faecalis from diseased tilapia. Int J Curr Microbiol Appl Sci. 2017;6(6):136–46.

[90] Hossain A, Habibullah-Al-Mamun M, Nagano I, Masunaga S, Kitazawa D, Matsuda H. Antibiotics, antibiotic-resistant bacteria, and resistance genes in aquaculture: risks, current concern, and future thinking. Environ Sci Pollut Res. 2022;29(8):11054–75. 10.1007/s11356-021-17825-4 35028843

[91] Xiong W, Sun Y, Zhang T, Ding X, Li Y, Wang M, Zeng Z. Antibiotics, antibiotic resistance Genes, and bacterial community composition in fresh water aquaculture environment in China. Microb Ecol. 2015;70(2):425–32. 25753824 10.1007/s00248-015-0583-x

[92] Ishida Y, Ahmed AM, Mahfouz NB, Kimura T, El-Khodery SA, Moawad AA, Shimamoto T. Molecular analysis of antimicrobial resistance in Gram-Negative bacteria isolated from fish farms in Egypt. J Vet Med Sci. 2010;72(6):727–34. 20145377 10.1292/jvms.09-0538

[93] Cabello FC. Heavy use of prophylactic antibiotics in aquaculture: a growing problem for human and animal health and for the environment. Environ Microbiol. 2006;8(7):1137–44. 16817922 10.1111/j.1462-2920.2006.01054.x

[94] Yuan X, Lv Z, Zhang Z, Han Y, Liu Z, Zhang H. A review of antibiotics, antibiotic resistant Bacteria, and resistance genes in aquaculture: Occurrence, Contamination, and transmission. In: Toxics 11; 2023. 10.3390/toxics11050420 PMC10223227 37235235

[95] Luthman O, Robb DHF, Henriksson PJG, Jørgensen PS, Troell M. Global overview of National regulations for antibiotic use in aquaculture production. Aquacult Int. 2024;32(7):9253–70.

[96] Meek RW, Vyas H, Piddock LJV. Nonmedical uses of antibiotics: time to restrict their use? PLoS Biol. 2015;13(10):e1002266. 26444324 10.1371/journal.pbio.1002266 PMC4621705

