# Supplementary material for: Characterization and antibiogram of bacterial isolates from diseased farmed Nile tilapia in Beheira governorate, Egypt
Source: BMC Vet Res. 2026 Jan 10;22:51. doi: 10.1186/s12917-025-05227-4 (PMC12849623; doi:10.1186/s12917-025-05227-4)
Supplement: Supplementary file 1 — Supplementary Material 1. [file 12917_2025_5227_MOESM1_ESM.docx]

**Table S1 (Supplementary Material):** Primer sets used for detection of virulence genes of recovered bacteria.

| Bacterial isolates | Virulence genes | Primers sequences  5'-3' | Amplified segment (bp) | Primary  denaturation | Amplification (35 cycles) | | | Final extension | References |
| --- | --- | --- | --- | --- | --- | --- | --- | --- | --- |
|  |  |  |  |  | Secondary denaturation | Annealing | Extension |  |  |
| *V. alginolyticus* | *Trh* | GGCTCAAAATGGTTAAGCG | 250 | 94˚C  5 min. | 94˚C  30 sec. | 54˚C  30 sec. | 72˚C  30 sec. | 72˚C  7 min. | (Mustapha et al., 2013) |
|  |  | CATTTCCGCTCTCATATGC |  |  |  |  |  |  |  |
|  | *Tdh* | CCATCTGTCCCTTTTCCTGC | 373 | 94˚C  5 min. | 94˚C  30 sec. | 54˚C  40 sec. | 72˚C  40 sec. | 72˚C  10 min. |  |
|  |  | CCAAATACATTTTACTTGG |  |  |  |  |  |  |  |
| *V. campbellii* | *tlh* | AAAGCGGATTATGCAGAAGCACTG | 449 | 94˚C  5 min. | 94˚C  30 sec. | 55˚C  40 sec. | 72˚C  45 sec. | 72˚C  10 min. | (Casandra et al., 2013) |
| *V. owensii* |  | GCTACTTTCTAGCATTTTCTCTGC |  |  |  |  |  |  |  |
| *A. veronii* | *Aerolysin* | CACAGCCAATATGTCGGTGAAG | 326 | 94˚C  5 min. | 94˚C  30 sec. | 52˚C  40 sec. | 72˚C  40 sec. | 72˚C  10 min. | (Singh et al., 2008) |
|  |  | GTCACCTTCTCGCTCAGGC |  |  |  |  |  |  |  |
|  | *act* | AGAAGGTGACCACCACCAAGAACA | 232 | 94˚C  5 min. | 94˚C  30 sec. | 55˚C  30 sec. | 72˚C  30 sec. | 72˚C  7 min. | (Nawaz et al., 2010) |
|  |  | AACTGACATCGGCCTTGAACTC |  |  |  |  |  |  |  |
| *E. faecalis* | *gelE* | TATGACAATGCTTTTTGGGAT | 213 | 94˚C  5 min. | 94˚C  30 sec. | 50˚C  30 sec. | 72˚C  30 sec. | 72˚C  7 min. | (Vankerckhoven et al., 2004) |
|  |  | AGATGCACCCGAAATAATATA |  |  |  |  |  |  |  |
|  | *cylA* | ACTCGGGGATTGATAGGC | 688 | 94˚C  5 min. | 94˚C  30 sec. | 50˚C  40 sec. | 72˚C  45 sec. | 72˚C  10 min. |  |
|  |  | GCTGCTAAAGCTGCGCTT |  |  |  |  |  |  |  |
| *S. agalactiae* | *Hyl* | CATACC TTAACAAAGATATATAACAA | 950 | 94˚C  5 min. | 94˚C  30 sec. | 52˚C  40 sec. | 72˚C  50 sec. | 72˚C  10 min. | (Krishnaveniet al., 2014) |
|  |  | AGATTTTTTAGAGAATGAGAAGTTTTTT |  |  |  |  |  |  |  |
|  | *cylE* | TGACATTTACAAGTGACGAAG | 248 | 94˚C  5 min. | 94˚C  30 sec. | 55˚C  30 sec. | 72˚C  30 sec. | 72˚C  7 min. | (Bergseng et al., 2007) |
|  |  | TTGCCAGGAGGAGAATAGGA |  |  |  |  |  |  |  |

**References: -**

- Bergseng, H., Bevanger, L., Rygg, M., & Bergh, K. (2007). Real-time PCR targeting the sip gene for detection of group B streptococcus colonization in pregnant women at delivery. Journal of Medical Microbiology, 56(2), 223-228.
- Krishnaveni, N., Isloor, S., Hegde, R., Suryanarayanan, V., Rathnma, D., Veeregowda, B., Nagaraja, C., & Sundareshan, S. (2014). Rapid detection of virulence associated genes in Streptococcal isolates from bovine mastitis. African Journal of Microbiology research, 8, 2245-2254.
- Mustapha, S., Mustapha, E. M., & Nozha, C. (2013). Vibrio alginolyticus: an emerging pathogen of foodborne diseases. International Journal of Science Technology, 2(4), 302-309.
- Nawaz, M., Khan, S. A., Khan, A. A., Sung, K., Tran, Q., Kerdahi, K., & Steele, R. (2010). Detection and characterization of virulence genes and integrons in Aeromonas veronii isolated from catfish. Food Microbiology, 27(3), 327-331.
- Singh, V., Rathore, G., Kapoor, D., Mishra, B. N., & Lakra, W. S. (2008). Detection of aerolysin gene in Aeromonas hydrophilla isolated from fish and pond water. Indian Journal of Microbiology, 48(4), 453-458.
- Vankerckhoven, V., Van Autgaerden, T., Vael, C., Lammens, C., Chapelle, S., Rossi, R., Jabes, D., & Goossens, H. (2004). Development of a multiplex PCR for the detection of asa1, gelE, cylA, esp, and hyl genes in Enterococci and survey for virulence determinants among European hospital isolates of Enterococcus faecium. Journal of Clinical Microbiology, 42(10), 4473-4479.

**TABLE S2 (Supplementary Material)**

Biochemical characteristics using VITEK 2 with 93% probability of *Vibrio alginolyticus*.

|  | | | | | | |  | | | | | | | | | | | |
| --- | --- | --- | --- | --- | --- | --- | --- | --- | --- | --- | --- | --- | --- | --- | --- | --- | --- | --- |
| 2 | APPA | + | 3 | ADO | - | 4 | | PyrA | + | 5 | IARL | - | 7 | dCEL | (-) | 9 | BGAL | - |
| 10 | H2S | - | 11 | BNAG | - | 12 | | AGLTp | - | 13 | dGLU | + | 14 | GGT | - | 15 | OFF | + |
| 17 | BGLU | - | 18 | dMAL | + | 19 | | dMAN | + | 20 | dMNE | + | 21 | BXYL | - | 22 | BAIap | - |
| 23 | ProA | + | 26 | LIP | - | 27 | | PLE | - | 29 | TyrA | + | 31 | URE | - | 32 | dSOR | - |
| 33 | SAC | - | 34 | dTAG | - | 35 | | dTRE | + | 36 | CIT | - | 37 | MNT | - | 39 | 5KG | - |
| 40 | ILATk | - | 41 | AGLU | - | 42 | | SUCT | - | 43 | NAGA | - | 44 | AGAL | - | 45 | PHOS | - |
| 46 | GlyA | + | 47 | ODC | - | 48 | | LDC | - | 53 | IHISa | - | 56 | CMT | + | 57 | BGUR | - |
| 58 | O129R | - | 59 | GGAA | + | 61 | | IMLTa | - | 62 | ELLM | - | 64 | ILATa | + |  |  |  |

**TABLE S3 (Supplementary Material)**

Biochemical Details using VITEK 2 with 50% probability of *Aeromonas sobria*.

|  | | | | | | |  | | | | | | | | | | | |
| --- | --- | --- | --- | --- | --- | --- | --- | --- | --- | --- | --- | --- | --- | --- | --- | --- | --- | --- |
| 2 | APPA | - | 3 | ADO | - | 4 | | PyrA | + | 5 | IARL | - | 7 | dCEL | - | 9 | BGAL | + |
| 10 | H2S | - | 11 | BNAG | - | 12 | | AGLTp | - | 13 | dGLU | + | 14 | GGT | - | 15 | OFF | - |
| 17 | BGLU | - | 18 | dMAL | + | 19 | | dMAN | - | 20 | dMNE | + | 21 | BXYL | - | 22 | BAIap | - |
| 23 | ProA | - | 26 | LIP | - | 27 | | PLE | - | 29 | TyrA | - | 31 | URE | - | 32 | dSOR | - |
| 33 | SAC | + | 34 | dTAG | - | 35 | | dTRE | + | 36 | CIT | - | 37 | MNT | - | 39 | 5KG | - |
| 40 | ILATk | - | 41 | AGLU | - | 42 | | SUCT | - | 43 | NAGA | - | 44 | AGAL | - | 45 | PHOS | - |
| 46 | GlyA | - | 47 | ODC | - | 48 | | LDC | - | 53 | IHISa | - | 56 | CMT | + | 57 | BGUR | - |
| 58 | O129R | + | 59 | GGAA | - | 61 | | IMLTa | - | 62 | ELLM | - | 64 | ILATa | - |  |  |  |

**TABLE S4 (Supplementary Material)**

VITEK 2 biochemical tests of *Enterococcus faecalis* (97% probability).

|  | | | | | | | | | | | | | | | | | |
| --- | --- | --- | --- | --- | --- | --- | --- | --- | --- | --- | --- | --- | --- | --- | --- | --- | --- |
| 2 | AMY | + | 4 | PIPLC | - | 5 | dXYL | - | 8 | ADH1 | + | 9 | BGAL | - | 11 | AGLU | - |
| 13 | APPA | - | 14 | CDEX | + | 15 | AspA | + | 16 | BGAR | - | 17 | AMAN | - | 19 | PHOS | - |
| 20 | LeuA | - | 23 | ProA | - | 24 | BGURr | - | 25 | AGAL | - | 26 | PyrA | + | 27 | BGUR | - |
| 28 | AlaA | + | 29 | TyrA | + | 30 | dSOR | + | 31 | URE | - | 32 | POLYB | + | 37 | dGAL | + |
| 38 | dRIB | + | 39 | ILATk | - | 42 | LAC | - | 44 | NAG | + | 45 | dMAL | + | 46 | BACI | + |
| 47 | NOVO | + | 50 | NC6.5 | + | 52 | dMAN | + | 53 | dMNE | + | 54 | MBdG | + | 56 | PUL | - |
| 57 | dRAF | - | 58 | O129R | + | 59 | SAL | + | 60 | SAC | + | 62 | dTRE | + | 63 | ADH2s | + |
| 64 | OPTO | + |  |  |  |  |  |  |  |  |  |  |  |  |  |  |  |


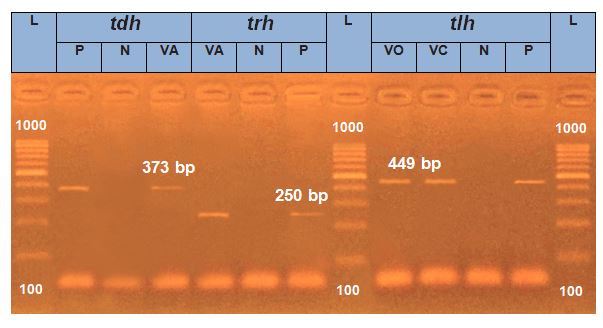


**Figure S1.** PCR amplification of the virulence genes of *Vibrio* strains isolated from diseased Nile tilapia (*Oreochromis niloticus*). Lane (**P**): the control positive sample; lane (**N**): the negative control sample; 100 bp DNA ladder; and lanes (**VA**): represent the *Vibrio* *alginolyticus* sample. The PCR products shown correspond to the predicted molecular mass of 373 bp (tdh gene) and 250 bp (trh gene). In addition to lanes (**VO**), represents the *Vibrio owensii* sample and (**VC**), represents the *Vibrio campbellii* sample. The PCR products shown correspond to the predicted molecular mass of 449 bp (tlh gene).


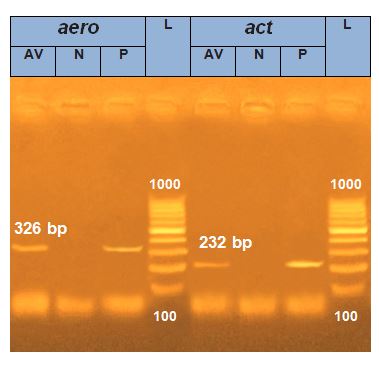


**Figure S2.** PCR amplification of the virulence genes of *Aeromonas veronii* strain isolated from diseased Nile tilapia (*Oreochromis niloticus*). Lane (**P**): the control positive sample; lane (**N**): the negative control sample; 100 bp DNA ladder; and lanes (**AV**): represent the *Aeromonas veronii* sample. The PCR products shown correspond to the predicted molecular mass of 326 bp (aerolysin gene) and 232 bp (cytotoxic enterotoxin gene).


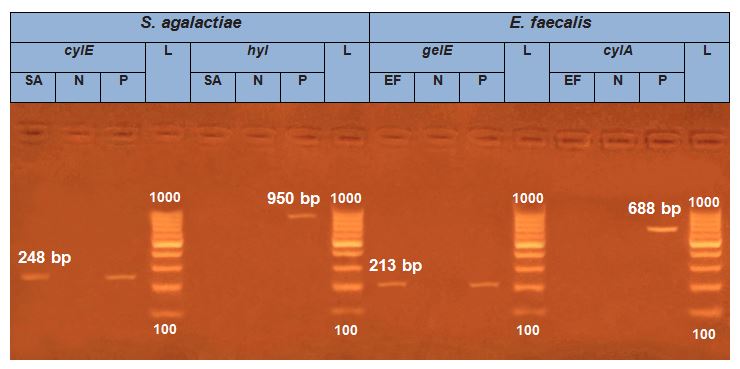


**Figure S3.** PCR amplification of the virulence genes of *Streptococcus agalactiae* and *Enterococcus faecalis* strains isolated from diseased Nile tilapia (*Oreochromis niloticus*). Lane (**P**): the control positive sample; lane (**N**): the negative control sample; 100 bp DNA ladder; and lanes (**SA**): represent the *Streptococcus agalactiae* sample. The PCR products shown correspond to the predicted molecular mass of 248 bp (β-hemolysincytolysin cylE gene) and negative for 950 bp (hyaluronidase hyl gene). In addition to lanes (**EF**), represents the *Enterococcus faecalis* sample. The PCR products shown correspond to the predicted molecular mass of 213 bp (gelatinase gelE gene), and negative for 688 bp (cytolysin cylA gene).
